# Protocol for CHAMPION study: a prospective study of maximal-cytoreductive therapies for patients with de novo metastatic hormone-sensitive prostate cancer who achieve oligopersistent metastases during systemic treatment with apalutamide plus androgen deprivation therapy

**DOI:** 10.1186/s12885-024-12395-3

**Published:** 2024-05-25

**Authors:** Beihe Wang, Jian Pan, Tingwei Zhang, Xudong Ni, Yu Wei, Xiaomeng Li, Bangwei Fang, Xiaoxin Hu, Hualei Gan, Junlong Wu, Hongkai Wang, Dingwei Ye, Yao Zhu

**Affiliations:** 1 Department of Urology, Fudan University Shanghai Cancer Center, No. 270 Dong’an Road, Shanghai, 200032 People’s Republic of China; 2 Shanghai Genitourinary Cancer Institute, Shanghai, China; 3 Department of Oncology, Shanghai Medical College, Fudan University, Shanghai, China; 4 Department of Radiology, Fudan University Shanghai Cancer Center, Shanghai, China; 5 Department of Pathology, Fudan University Shanghai Cancer Center, Shanghai, China

**Keywords:** Hormone-sensitive prostate cancer, Apalutamide, Prostate-specific membrane antigen, Positron emission tomography/computed tomography, Radical prostatectomy

## Abstract

**Background:**

The proposed trial is to examine the feasibility of prostate-specific membrane antigen (PSMA) positron emission tomography/computed tomography (PET/CT)-guided cytoreduction plus apalutamide and androgen deprivation therapy (ADT) for newly diagnosed metastatic hormone-sensitive prostate cancer (mHSPC) at oligometastatic state.

**Methods:**

CHAMPION (NCT05717582) is an open-label, single-arm, phase II trial, planning to enroll newly diagnosed mHSPC cases with oligometastases (≤ 10 distant metastatic sites in conventional imaging). Patients will receive 6 cycles of apalutamide plus ADT. Patients with oligometastatic disease at PSMA PET/CT after 3 treatment cycles will receive cytoreductive radical prostatectomy. PSMA PET/CT-guided metastasis-directed external radiation therapy will be determined by the investigators. Apalutamide plus ADT will be continued for 2 weeks postoperatively. The primary endpoint is the proportion of patients with undetectable prostate-specific antigen (PSA), no disease progression, and no symptom deterioration after 6 cycles of apalutamide plus ADT. Secondary endpoints include the percentage of patients with PSA ≤ 0.2 ng/mL and oligometastases by the end of 3 treatment cycles, PSA response rate, and safety. Fleming’s two-stage group sequential design will be adopted in the study, where the null hypothesis is that the rate of patients with an undetectable PSA is ≤ 40% after 6 cycles of treatment, while the alternate hypothesis is an undetectable PSA of > 60%; with one-sided α = 0.05, power = 0.80, and an assumed dropout rate of 10%, the required number of patients for an effective analysis is 47. Enrolment in the study commenced in May 2023.

**Discussion:**

The multi-modal therapy based on treatment response may improve the prognosis of newly diagnosed mHSPC patients with oligometastases.

**Trial registration:**

The study is registered with Clinical Trials.Gov (NCT05717582). Registered on 8th February 2023.

**Supplementary Information:**

The online version contains supplementary material available at 10.1186/s12885-024-12395-3.

## Introduction

Prostate cancer is a lethal disease especially when progressing into a metastatic status [1, 2]. The proportion of patients with newly diagnosed prostate cancer at advanced stages is higher in China than in Western countries, and most patients have distant metastases diagnosis [3]. Currently, novel hormone therapy (NHT) plus androgen deprivation therapy (ADT) has become the standard first-line treatment for patients with metastatic hormone-sensitive prostate cancer (mHSPC) [4]. However, the 5-year overall survival (OS) for patients receiving this regime remained unsatisfying [5]. Treatment strategies should be refined to improve the prognosis of mHSPC.

Oligometastatic prostate cancer is an intermediate phase between a primary localized state and an extensive metastatic state and differs from widely metastatic lesions. The dynamic response to therapeutic treatment emphasizes the importance of this treatment window to defer disease progression [6, 7]. The definition of oligometastatic disease is ambiguous in prostate cancer with less than 3, 5, or 10 metastatic sites and free of visceral metastasis [8–15]. A phase II clinical trial demonstrated that the addition of local treatment (mostly cytoreductive radical prostatectomy [cRP], accounting for 85%) to systemic therapy significantly prolonged radiographic progression-free survival (rPFS) and OS in mHSPC patients with ≤ 5 metastases [16]. Recent evidence has showed survival benefits and cost-effectiveness for multi-modal therapy (MMT) in mHSPC patients with limited metastatic lesions [17, 18]. Besides systemic therapy, MMT strategy further included local treatment (e.g., cRP and external beam radiotherapy for prostate) and metastasis-directed therapy (MDT, e.g., stereotactic body radiation therapy [SBRT]) [19–21]. However, current MMT evidence mostly include traditional ADT instead of NHT. The benefit of NHT in MMT strategy should be further evaluated.

Prostate-specific membrane antigen (PSMA) positron emission tomography/computed tomography (PET/CT) has displayed diagnostic accuracy in prostate cancer [22]. Although, more metastatic lesions could be identified by PSMA PET/CT, the migration from low- to high-volume disease only occurred in 22% of patients according to the CHAARTED criteria [23]. Therefore, the value of PSMA PET/CT for newly diagnosed mHSPC still needs to be evaluated by its application during natural disease course. In a prospective trial, about 63% of newly diagnosed oligometastatic prostate cancer cases had a complete radiological response to systemic therapy plus pelvic lymph node dissection [PLMD] guided by PSMA PET/CT and 47% of cases had a pathological complete response, suggesting the potential value of PSMA PET/CT in treatment evaluation and decision-making [24]. Furthermore, a previous study by our group found that about 50% of nonmetastatic castration-resistant prostate cancer on conventional imaging has been proved to be metastatic on PSMA PET/CT, and the incidence of metastatic disease was less frequent in patients who received SBRT compared with those who did not [25].

These results suggested that when systemic therapy had inhibited the function of most distant metastases, the functional residual lesions might need individualized MDT. MMT strategies based on treatment response evaluated by PSMA PET/CT might be optimal for managing mHSPC with oligometastatic state.

### Trial overview

**Fig. 1:**
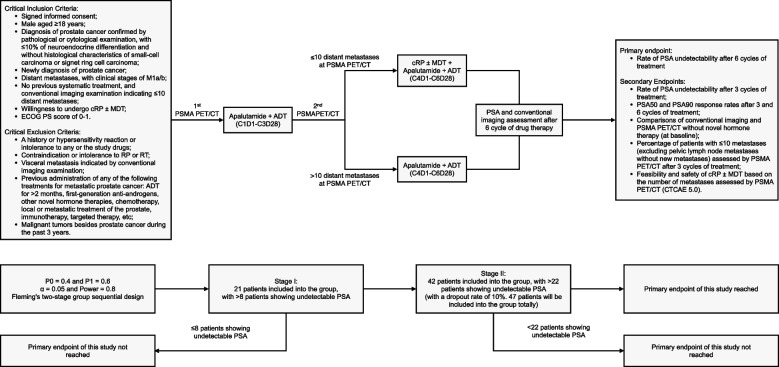
The flowchart of the proposed trial. MDT: metastasis-directed therapy; cRP: cytoreductive radical prostatectomy; ECOG PS: Eastern Cooperative Oncology Group Performance Status; RT: radiation therapy; ADT: androgen deprivation therapy; PSMA-PET/CT: prostate-specific membrane antigen positron emission tomography/computed tomography; PSA: prostate-specific antigen; CTCAE: Common Terminology Criteria for Adverse Events

This study is a prospective, single-arm, open-labeled, phase II clinical trial that will be conducted from March 2023 to December 2024 at Fudan University Shanghai Cancer Center (FUSCC). Patients with newly diagnosed metastatic mHSPC and ≤ 10 distant metastatic sites will be enrolled for the study. The patients will receive 6 cycles of apalutamide in combination with ADT. Gallium-68 (^68^Ga) or fluorine-18 (^18^F) PSMA PET/CT will be used for viewing metastatic sites after 3 treatment cycles. In patients with metastatic oligoresidues (≤ 10 distant metastases), cRP will be performed. During prostatectomy, ADT will be maintained. Apalutamide plus ADT will be continued for 2 weeks after surgery, and PSMA PET/CT-guided metastasis-directed SBRT will be adopted as determined by the investigators (Fig. 1). This study protocol will follow the Good Clinical Practice standards and the Declaration of Helsinki principles. This prospective study has been approved by the Clinical Research Ethics Committee of FUSCC (number 1909207–12) and has been registered with ClinicalTrials.gov (NCT05188911). The written informed consent will be obtained from all participants.

The primary endpoint is the proportion of patients with undetectable prostate-specific antigen (PSA), no disease progression, and no symptomatic deterioration after 6 treatment cycles. Undetectable PSA is defined as ≤ 0.2 ng/mL and confirmed by two successive evaluations at least 3 weeks apart. Disease progression will be evaluated by a blinded independent central review (ICR) according to the Response Evaluation Criteria in Solid Tumours (RECIST) version 1.1. Symptomatic deterioration is referred to as a situation where the treatment needs to be interrupted, but with no apparent disease progression.

The secondary endpoints include the following:

1. Percentage of cases with undetectable PSA after 3 treatment cycles.
2. PSA50 and PSA90 response rate after 3 and 6 treatment cycles. PSA50 and PSA 90 response rate are the percentages of patients who have a reduction of greater than 50% and 90% from baseline, respectively.
3. Comparisons of baseline conventional imaging and PSMA PET/CT imaging features, including tumor-node-metastasis (TNM) staging, molecular imaging TNM (miTNM) classification, number of metastases, and metastatic sites.
4. Proportion of patients with ≤ 10 metastases (except pelvic lymph node metastases without new metastases) assessed by PSMA PET/CT at the end of cycle 3.
5. Comparison of conventional imaging and PSMA PET/CT imaging features at the end of the 3 and 6 treatment cycles, including change in prostate volume, number and volume of prostate tumor nodules, distribution of metastatic lesions and number of metastatic lesions.
6. Safety assessment: including adverse events (graded according to National Cancer Institute's Common Terminology Criteria for Adverse Events 5.0 criteria) and surgical complications (graded according to Clavien-Dindo classification).
7. Assess the feasibility of implementing cRP ± MDT based on the number of metastases in PSMA PET/CT.

The exploratory endpoints include the following:

1. SUVmax and tumor volume in PSMA PET/CT after 3 treatment cycles, and their correlations to PSA response rate and demographic characteristics.
2. Subgroup analysis stratified by baseline SUVmax and number of metastases.
3. Pathological complete response (defined as the percentage of patients having no surviving tumor cells in prostatectomy specimens following preoperative treatment), minimal residual disease (defined as residual tumor ≤ 5 mm in prostatectomy specimens following preoperative treatment), and pathological tumor-node.
4. Time to PSA progression, defined as the time from the beginning of treatment to the first PSA progression based on Prostate Cancer Working Group 2 criteria.
5. rPFS, defined as the time from treatment initiation until radiographic progression based on RECIST 1.1 criteria and death from any cause.
6. Metastatic progression pattern in patients achieving a minimal residual state after treatment.
7. Natural course of disease in patients with multiple metastases before and after treatment.
8. Biomarker search: prostate cancer-related molecular markers.

## Participants and methods

This study will enroll patients with newly diagnosed mHSPC and distant metastases of ≤ 10 detected by conventional imaging. All patients will receive apalutamide plus ADT.

### Inclusion criteria

Participants who satisfy all the following will be eligible for the study.

- Able to understand and willing to sign the informed consent.
- Men aged ≥ 18 years.
- With histologically or cytologically confirmed prostate adenocarcinoma (primary small cell carcinoma or signet-ring cell carcinoma of the prostate are not allowed, however adenocarcinoma with neuroendocrine differentiation accounting ≤ 10% is allowed).
- Newly diagnosed prostate cancer (within 3 months prior to enrollment).
- With distant metastatic disease (M1a/b staging) assessed via conventional imaging including bone imaging, conventional CT, or magnetic resonance imaging [MRI].
- With ≤ 10 distant metastatic sites assessed via conventional imaging before systemic treatment.
- Receipt of apalutamide plus ADT as first-line treatment is the next treatment option. Prior therapy with ADT or first-generation antiandrogen agent (e.g., bicalutamide, flutamide) or apalutamide + ADT for ≤ 2 months before enrollment are permitted.
- Receipt of PSMA PET/CT within 6 weeks before receiving apalutamide plus ADT and without prior treatment of NHT or chemotherapy before PSMA PET/CT examination.
- Willing to accept cRP and/or PLMD ± MDT.
- With Eastern Cooperative Oncology Group performance status of 0–1.
- With adequate organ function.
- With life expectancy ≥ 12 months.

### Exclusion criteria

Participants with any of the following will be excluded from the study.

- History of hypersensitivity reaction or intolerance to any drug involved in the study.
- Contraindication or intolerance to cRP or radiation therapy
- With visceral metastasis assessed via conventional imaging.
- Prior treatment with any of the following: ADT or first-generation antiandrogen agent (such as bicalutamide and flutamide) for > 2 months; other NHT (such as abiraterone, enzalutamide, and darutamide); chemotherapy; any form of local prostate therapy, including surgery or RT (such as external beam radiotherapy [EBRT], SBRT, brachytherapy, and radiopharmaceutical therapy); any form of metastatic treatment, including surgery or RT (such as EBRT, SBRT, brachytherapy, and radiopharmaceutical therapy), but prior transurethral resection of the prostate for benign prostatic hyperplasia is permitted; immunotherapy; targeted therapy.
- History of seizures, medication that lowers the seizure threshold, or a seizure-inducing illness within 12 months before starting study treatment (including history of transient ischemic attack, stroke, and traumatic brain injury requiring hospitalization with disturbance of consciousness).
- Receipt of major surgery within 4 weeks before starting study treatment.
- With major cardio-cerebrovascular disease within 6 months before study treatment, including severe/unstable angina, myocardial infarction, congestive heart failure [New York Heart Association class III or IV], cerebrovascular accident, or arrhythmia requiring medical treatment.
- With swallowing disorder, chronic diarrhea, intestinal obstruction, or other factors affecting drug ingestion and absorption.
- With active infection, such as serologically positive for human immunodeficiency virus (HIV), positive HBV surface antigen (HBsAg) result, and positive for hepatitis C virus (HCV) antibody, which may affect the safety and efficacy of medication according to the investigator's judgment.
- Other malignancy within the last 3 years or at the same time, adequately treated non-melanoma skin cancer is permitted.
- With known brain metastases or active meningitis.
- Participation in another clinical study with investigational or medical devices.
- Unable to cooperate with treatment and follow-up procedures.
- With concomitant diseases (such as poorly controlled hypertension, severe diabetes, neurological, and psychiatric diseases) or any other condition that could seriously compromise patient safety, confound the study results, or prevent patients from completing the study.

### Study treatment

Patients will receive apalutamide in combination with ADT. Each treatment cycle will be 28 days, with a total of 6 treatment cycles. Apalutamide will be given orally at a dosage of 240 mg once daily, but this dose will be adjusted according to the instruction in case of intolerability [26]. The ADT regimen consists of a gonadotropin-releasing hormone analog (GnRHa), either a GnRHa agonist or a GnRHa antagonist. The type, frequency, and dosage of the ADT will be determined by the investigators, and the dosage will be adjusted according to the instruction if adverse events occur.

The PSMA PET/CT imaging will be performed in a 2-week window after 3 cycles of apalutamide plus ADT. Based on the PSMA PET/CT images, the investigators will decide on subsequent cytoreduction. The patients with ≤ 10 distant metastatic sites by PSMA PET/CT, will be treated with cRP with/without MDT. cRP and PLND will be performed based on the evaluation of preoperative PSMA PET/CT images. ADT will be maintained during the whole perioperative period, while apalutamide will be discontinued for at least 1 week before the day of surgery. Apalutamide plus ADT will be restored 2 weeks postoperatively based on the evaluation by investigators. A multidisciplinary team including investigators will reach a decision on the application of MDT. SBRT will be performed and the dosages for different body sites will vary based on the tolerance level of the organ with metastases and the surrounding normal tissue. The lesions will be exposed to radiation with a recommended protocol using guidelines and the clinical experience of the investigators. The patients will be concurrently administered apalutamide plus ADT during the radiotherapy. For patients undergoing cytoreduction (cRP with/without MDT), the entire treatment duration will not exceed 8 months.

Cases recovering from the oligometastatic state after three cycles of apalutamide plus ADT assessed by PSMA PET/CT imaging will continue to receive apalutamide plus ADT. Treatment will be terminated if the patients cannot benefit from the therapy, experience intolerant toxicity, or withdraw the informed consent. For patients who discontinue apalutamide treatment plus ADT within the first three months of the trial, subsequent treatment will be determined by the investigators.

### Assessments

Serum PSA will be assessed, and PSMA PET/CT imaging will be performed once every 3 cycles during the systemic treatment [27]. If serum PSA level increases or patients suffer from osteodynia, chest, abdominal, and pelvic CT/MRI imaging will be performed. The participants will be followed up via telephone until the end of the study, death, or loss to follow-up, within ± 4 weeks (Table 1). ^68^Ga or ^18^F can be used as a tracer and the type of tracer for each patient will remain unchanged throughout the study.

**Table 1:** Participant timeline

	Screening Period		Observation and Treatment Period											Group Exit Confirmation	Follow-up Period
Item	Within 14 days before first dosing	Within 7 days before first dosing	1st cycle	2nd cycle	3rd cycle		Planned surgery date (First 7 ± 1 days)	Surgery date	After surgery date (14 ± 3 days)	4th cycle	5th cycle	6th cycle	6th cycle	Group-exit investigation & observation confirmation	Safety & survival follow-up
			C1D1 (± 3 days)	C2D1 (± 3 days)	C3D1 (± 3 days)	C3D28 (± 3 days)				C4D1 (± 3 days)	C5D1 (± 3 days)	C6D1 (± 3 days)	C6D28 (± 3 days)	C6D28 + 30 (± 7 days)	Once every 3 m in 1st–2nd y; once every 6 m in 3rd y
Signing of informed consent [1]	√														
Demographic characteristics and history of tumor or other diseases [2]	√														
Vital signs [3]		√				√	√						√	√	
Physical examination [4]		√													
ECOG scoring [5]		√				√							√	√	
Testosterone examination [6]	√														√
Blood routine [7]		√													
Urine routine [8]		√													
Occult blood in stool [9]		√													
Blood biochemistry [10]		√													
Blood coagulation function [11]		√													
HIV, Hepatitis B, Hepatitis C [12]	√														
12-lead electrocardiogram [13]		√													
Ultrasonic cardiogram [14]	√														
Prostate specimen examination [15]	√							√							
PSA examination [16]		√				√							√	√	√
Imaging examination [17]	√												√		√
Bone scan [18]													√		√
PSMA-PET [19]	√					√									√
Metastasis-directed therapy [20]										√					
Systemic therapy drug compliance [21]						√							√	√	
Concomitant drug/therapy [22]	√													√	
Adverse event recording [23]						√							√	√	
Progressing event, survival state, and subsequent tumor treatment [24]														√	√

Description:

a: Medical and treatment history, including present and past medical history, history of surgery and allergy, and history of anticancer therapy (chemotherapy/radiotherapy/targeted therapy/hormone therapy/surgery)

b: Vital signs, including temperature, respiration, pulse, and blood pressure

c: Physical examination, including height and weight measurements, and assessments of the general body surface, skin, head and neck, chest, abdomen, limbs, nervous system, and lymph nodes. Height will be measured only in the screening period, not during other visits and interviews

d: Laboratory examinations:

➢ In case the interval between the laboratory examination and the examination on the 1^st^ day of the 1^st^ week in the screening period is less than 7 days, the examination will not be performed on the first day of the first week

➢ Blood routine test evaluating red blood cell (RBC), white blood cell (WBC), hemoglobin (HGB), platelet (PLT), and differential counts of white blood cells (basophil count, BASO#; eosinophil count, EO#; lymphocyte count, LYMPH#; monocyte count, MONO#; neutrophil count, NEUT#; neutrophil percentage, NEUT%)

➢ Blood biochemical analysis detecting alanine aminotransferase (ALT), aspartate aminotransferase (AST), alkaline phosphatase (ALP), glutamyl transpeptidase (GGT), total bilirubin (TBIL), urea nitrogen (BUN)/urea (UREA), serum creatinine (SCr), potassium (K), sodium (Na), chlorine (Cl), magnesium (Mg), calcium (Ca), phosphorus (P), glucose (GLU), triglyceride (TG), total cholesterol (TC), low-density lipoprotein (LDL-C), high-density lipoprotein (HDL-C), and albumin (ALB)

➢ Urine routine detecting urine pH, urine protein (U-PRO), urine glucose (U-GLU), urinary white blood cell (U-WBC), and urinary red blood cell (U-RBC)/hematuria

➢ Coagulation function (during screening and at the end of treatment) will be assessed by determining prothrombin time (PT), activated partial thromboplastin time (APTT), fibrinogen (FIB), thrombin time (TT), and international normalized ratio (INR)

e: Prostate specimen examination: Puncture biopsy specimen, liquid biopsy specimen, or paraffin section specimen of the prostate will be collected to detect prostate-cancer-related biomarkers

f: After 6 cycles of treatment, the investigators will determine if PSMA PET/CT examination is needed

g: After 3 cycles of treatment, a patient with elevated serum PSA level and ostalgia will undergo conventional imaging examination (bone scan, and CT/MRI of the chest, abdomen, and pelvic cavity)

h: Adverse events and drug combinations: From the signing of informed consent to 30 days after the last study drug administration, the adverse events (including severe adverse events) and drug combinations will be recorded

### Data management

This trial will use case report forms (CRF) to collect basic and clinical data. The investigators will maintain source files for each subject, including original medical records and other written data or records. The data entered from the CRF must be linked to source files for each subject. Data managers will write the data review reports following the trial protocol and data review criteria from databases. Primary investigators, statisticians, and data managers will attend the data review meetings, and the data will be reviewed, resolved, and signed by the representatives attending the meetings. After all personnel approve the data, data managers will organize and execute data-locking tasks. The locked data will be submitted to statisticians for statistical analysis.

### Monitoring

An independent data and safety monitoring committee will be established to assess safety when serious adverse events occur. All adverse events will be recorded. A qualified independent auditor will be appointed to scrutinize all trial protocols, and the trial will be conducted before and during the treatment period based on a written procedure.

### Statistical analysis

The primary endpoint is the rate of participants with an undetectable PSA after 6 cycles of the apalutamide plus ADT. Fleming's two-stage group sequential design will be employed in this study, where the null hypothesis is that the rate of participants with an undetectable PSA will be ≤ 40%, and the alternate hypothesis was an undetectable PSA of > 60%. The test will be one-sided, with an α of 0.05 and a power value of 0.80. The first stage of the study will enroll 21 patients; if the number of patients with undetectable PSA is ≤ 8 during this stage, this treatment strategy will be considered ineffective and the study will be terminated. In case the number of patients with undetectable PSA is > 8 at the initial stage, this study will enter its second stage, when more patients will be added to reach the sample size of 42. If the number of patients with undetectable PSA is ≤ 22 during this period, this protocol will be considered ineffective. With an assumption of a dropout rate of 10%, a total of 47 patients will be needed for the effective analysis.

The full analysis set (FAS) will be used for efficacy analysis. The FAS follows the intend-to-treat (ITT) principle, where patients receiving at least one treatment are included in the analysis. The safety analysis set will include all patients receiving at least one treatment in this study, using the safety records after the treatment.

Quantitative data with normal distribution or near normal distribution will be expressed as mean ± standard deviation; those with skewed distribution or heterogeneity of variance will be expressed as median and quartile. Categorical data will be expressed as numbers and percentages. Categorical data will be analyzed by chi-square test or Fisher's exact test. The Kaplan–Meier method will be used to calculate the median times (95% confidence intervals) for time-to-event variables and generate survival plots. Each variable will be analyzed against the baseline value of the screening period. Paired t-test, χ2-test, exact probability method, or non-parametric test will be utilized to assess the differences of determinants before and after the study.

### Study status

The CHAMPION study commenced patient enrollment in March 2023 and is currently ongoing with patient recruitment.

## Discussion

Metastatic prostate cancer is a major cause of mortality among men [1]. The proportion of patients with advanced prostate cancer when first diagnosed and treatment is higher in China compared to other developed counties [3]. The 5-year OS rate of patients with newly diagnosed metastatic prostate cancer is only about 32.3% [5]. In metastatic prostate cancer, the oligometastatic state (low-tumor-burden disease state) differs in biological characteristics, clinical manifestations, and response to therapeutic intervention, compared to the advanced metastatic state, providing a good treatment window to prevent/slow disease progression [6, 7]. Therefore, developing effective treatment strategies for prostate cancer with oligometastatic state would be beneficial to many patients with metastatic prostate cancer.

MMT consists of local therapeutic intervention and systemic treatment and is recommended for low-volume mHSPC [17, 18]. Investigators at John Hopkins University first proposed total eradication therapy (TET) in 2019. The MMT protocol for TET comprised systematic treatment, local therapeutic intervention, and MDT, and preliminarily demonstrated the feasibility and efficacy in managing metastatic prostate cancer. The HORRAD and STAMPEDE clinical trials also confirmed the benefits of combined treatment with local therapeutic intervention and systemic treatment in mHSPC patients [21, 28]. Systematic treatment mostly included conventional ADT; currently, the NHT containing apalutamide plus ADT has become the standard treatment option for mHSPC. However, the effect of this combined therapy in patients with low-tumor-burden mHSPC still needs to be explored.

Limitations on the number of distant metastases that determines whether patients should undergo MMT or not continuously vary with changes in treatment techniques and notions. In the STAMPEDE trial, the survival benefits and failure-free survival were dependent on the number of bone metastases based on the subgroup analysis [21]. In patients with ≤ 4 bone metastases, the OS was significantly prolonged after radiation therapy; patients with 4–8 bone metastases still benefited from the radiation therapy; patients with as many as 9 bone metastatic sites benefited only from failure-free survival. Assessing SBRT in patients with 4–10 metastases, a phase III clinical study (SABR-COMET-10) found SBRT feasible and effective in patients with pan-tumor oligometastases, including 4–10 prostate cancer oligometastases [14]. This proposed trial will incorporate patients with newly diagnosed mHSPC and ≤ 10 metastatic sites to determine the feasibility and potential benefit of MMT.

Furthermore, identifying the suitable lesion and selecting the optimal timing and in MDT treatment remains controversial. Since PSMA PET/CT has higher diagnostic accuracy than MRI, CT, and prostate ultrasonography it might serve as an effective evaluation method during follow-up [29–31]. Therefore, this study will evaluate the feasibility of using PSMA PET/CT and its diagnostic accuracy to guide decision-making for cytoreduction in patients with newly diagnosed mHSPC treated with apalutamide and ADT.

This study will have a few limitations to be considered. First, this is a single-arm clinical trial without comparators. Therefore, the benefit of the treatment strategies in this study should be verified in further studies. Secondly, this is an exploratory trial with a relatively small sample size, and a firm conclusion cannot be drawn. Nevertheless, the findings of this study will provide preliminary evidence for a large randomized controlled clinical trial.

In conclusion, this study will provide a new clinical strategy, consisting of NHT and cytoreduction guided by PSMA PET/CT-guided in treating patients with newly diagnosed mHSPC and ≤ 10 metastatic sites. Further, the feasibility of the individualized therapeutic intervention developed in this study, including systemic therapy, cRP and MDT will be explored for managing patients with mHSPC. The findings of this study might provide evidence of the clinical management pathway, to further improve prognosis in patients with mHSPC.

### Supplementary Information

Supplementary Material 1.

## Data Availability

No datasets were generated or analysed during the current study.

## Abbreviations

ADT: Androgen deprivation therapy
CRF: Case report forms
cRP: Cytoreductive radical prostatectomy
EBRT: External beam radiotherapy
FAS: Full analysis set
GnRHa: Gonadotropin-releasing hormone analog
ICR: Independent central review
ITT: Intend-to-treat
MDT: Metastasis-directed therapy
mHSPC: Metastatic hormone-sensitive prostate cancer
MMT: Multi-modal therapy
MRI: Magnetic resonance imaging
NHT: Novel hormone therapy
OS: Overall survival
rPFS: Radiographic progression-free survival
SBRT: Stereotactic body radiation therapy
PET/CT: Positron emission tomography/computed tomography
PLND: Pelvic lymph node dissection
PSA: Prostate-specific antigen
PSMA: Prostate-specific membrane antigen
RECIST: Response Evaluation Criteria in Solid Tumours
TET: Total eradication therapy
TNM: Tumor-node-metastasis
